# Baseline Characteristics of fall from Height Victims Presenting to Emergency Department; a Brief Report 

**Published:** 2017-01-31

**Authors:** Hamidreza Hatamabadi, Ali Arhami Dolatabadi, Batoul Atighinasab, Saeed Safari

**Affiliations:** 1 Safety Promotion and Injury Prevention Research Center, Shahid Beheshti University of Medical Sciences, Tehran, Iran.; 2 Emergency Department, Imam Hossein Hospital, Shahid Beheshti University of Medical Sciences, Tehran, Iran.; 3 Emergency Department, Emdade Shahid Beheshti Hospital, Sabzevar University of Medical Sciences, Sabzevar, Iran.; 4 Emergency Department, Shohadaye Tajrish Hospital, Shahid Beheshti University of Medical Sciences, Tehran, Iran.

**Keywords:** Accidental falls, multiple trauma, emergency service, hospital, population characteristics, patient outcome assessment

## Abstract

**Introduction:**

Trauma due to accidents or fall from height is a major cause of disability and mortality. The present study was designed aiming to evaluate the baseline characteristics of fall from height victims presenting to emergency department (ED).

**Methods:**

This prospective cross-sectional study evaluates the baseline characteristics of fall from height cases presenting to EDs of three educational Hospitals, Tehran, Iran, during one year. Data were analyzed using SPSS 21 and presented using descriptive statistics.

**Results:**

460 patients with the mean age of 27.89 ± 20.95 years were evaluated (76.5% male). 191 (41.5%) falls occurred when working, 27 (5.9%) during play, and 242 (52.6%) in other times. Among construction workers, 166 (81.4%) had not used any safety equipment. Fracture and dislocation with 180 (39.1%) cases and soft tissue injury with 166 (36.1%) were the most common injuries inflicted. Mean height of falling was 3.41 ± 0.34 (range: 0.5 – 20) meters. Finally, 8 (1.7%) of the patients died (50% intentional) and 63% were discharged from ED. A significant correlation was detected between mortality and the falls being intentional (p < 0.0001) as well as greater height of fall (p < 0.0001).

**Conclusion:**

Based on the findings, most fall from height victims in the present study were young men, single, construction workers, with less than high school diploma education level. Intentional fall and greater height of falling significantly correlated with mortality.

## Introduction

Trauma due to accidents or fall from height is a major cause of disability and mortality among individuals aged less than 45 years in the United States of America, Europe and third world countries (1). The cost of treatment and rehabilitation of these patients is estimated to be more than 33 billion dollars in the United States of America (2, 3). Prevention is the most important solution for facing this phenomenon, which requires knowing the epidemiology of trauma (4-7). Falling from height, happens at least once for about one third of housekeepers over 65 years old (8, 9). In a study by Amani et al., fall from height was among the most common trauma mechanisms in their studied population with 38.5% prevalence (6). Usually, the elderly are affected with this type of trauma following every-day activities and adults face it due to occupational accidents (10). In a study by Zamani et al., evaluating hospitals of Isfahan, fall from height was the second most common trauma mechanism after traffic accidents (7). Fall from height has been the major cause of trauma related hospitalization for both male and female individuals in Ireland (11). Based on the statistics by the United States department of labor, approximately 150000 construction accidents happen annually. Any injuries after falling can cause serious and permanent disabilities and increase the patient’s need for receiving treatment (12). Falls from more than 6 meters are usually counted as high-energy trauma and are referred to trauma centers (13). Identifying the epidemiologic characteristics of fall from height victims can be beneficial in prevention and treatment of these patients. Therefore, the present study was designed aiming to evaluate the baseline characteristics of fall from height victims presenting to emergency department (ED).

## Methods

***Study design and setting***

The present study is a prospective cross-sectional one that evaluates the baseline characteristics of fall from height cases in patients presenting to ED of Imam Hossein, Shohadaye Tajrish, and Loghmane Hakim Hospitals, Tehran, Iran, from March 2012 to March 2013. Protocol of the present study was approved by the ethics committee of Shahid Beheshti University of Medical Sciences and in agreement with the Declaration of Helsinki, the researchers adhered to protecting patients’ privacy and keeping their information confidential. Informed consent form was signed by the patients before participating in the study. 

***Participants***

Census method was used for enrolling the participants and all victims of fall from height presenting to the 3 mentioned hospitals during one year were evaluated. No age or sex limitation was considered for this study.

***Data gathering***

By referring to the clinical profile and/or directly interviewing the patient or their relatives a checklist including demographic data (age, sex, marital status, occupation, level of education), route of transportation (ambulance or private vehicle), being intentional or unintentional, falling location (staircase, scaffold, rooftop, ladder), presence or absence of safety equipment, and outcome (discharge from ED, transfer to another hospital, admission to either surgery or neurosurgery ward, death) was filled for every patient. Data were gathered by a senior emergency medicine resident under supervision of an emergency medicine specialist.

***Statistical analysis***

Data were analyzed using SPSS statistical software version 21. For reporting quantitative variables mean ± standard deviation (SD) was used and for qualitative findings, frequency and percentage were described. Chi-square or Fisher’s exact tests were used for comparing qualitative variables and t-test was applied for comparing means. P < 0.05 was considered as significance level.

## Results

Demographic data

460 patients with the mean age of 27.89 ± 20.95 years (2 months – 94 years) were evaluated (76.5% male). 413 (89.8%) cases presented to Imam Hossein Hospital, 26 (5.7%) to Shohadaye Tajrish Hospital, and 21 (4.6%) to Loghmane Hakim Hospital. Table 1 shows the baseline characteristics of the patients. 191 (41.5%) falls occurred when working, 27 (5.9%) during play, and 242 (52.6%) in other times. Mean time to ambulance arrival was 16.9 ± 9.26 minutes and mean time to reach hospital was 27.11 ± 16.7 minutes. Among construction workers, 166 (81.4%) had not used any safety equipment. Table 2 depicts clinical findings of the patients on arrival. Fracture and dislocation with 180 (39.1%) cases and soft tissue injury with 166 (36.1%) were the most common injuries inflicted. Mean height of falling was 3.41 ± 0.34 (range: 0.5 – 20) meters.

***Outcome***

Table 3 shows the ED outcome of the patients. Finally, 8 (1.7%) of the patients died, 4 (50%) of which were intentional cases. Mean height of falling was 6.77 ± 4.11 meters for intentional cases, which was significantly higher than unintentional accidents with the mean height of 3.23 ± 2.96 (p < 0.0001). A significant correlation was detected between mortality and the falls being intentional (p < 0.0001) as well as greater height of fall (p < 0.0001). 

## Discussion

Based on the findings, most fall from height victims in the present study were young men, single, construction workers, with less than high school diploma education level who were presented to ED following fall from a staircase or scaffold. 63% of the patients were discharged from ED after receiving diagnostic and treatment services and reaching full recovery, while 1.7% of them died. Intentional fall and greater height of falling significantly correlated with mortality.

In a study by Salimi et al. on 1141 trauma patients in Ahwaz, 83.4% of the trauma patients were male (14). Studies in Ardabil and Isfahan provinces also showed that about 70% of fall from height patients were male (6, 15). The reason for the high male to female ratio could be that construction workers are mostly men and they are more at risk of falling due to the type of their activity. In this study, 57.4% of the patients had a mean age of 27 years. In other words, most trauma patients were young individuals less than 30 years old, which is in line with findings of numerous other studies (6, 14, 15). In the current study, about 150 patients were under 18 years old. Most children had falling accidents by falling from the arms of their parents or as a result of their neglect. Therefore, by paying more attention and taking safety measures, these accidents can be prevented.

**Table 1:** Baseline characteristics of studied patients

**Variable**	**Number (%)**
**Age (year)**	
< 18	150 (32.6)
18 - 60	246 (57.4)
> 60	46 (10)
**Sex**	
Male	352 (76.5)
Female	108 (23.5)
**Marital Status**	
Married	197 (42.8)
Single	263 (57.2)
**Occupation**	
Construction worker	204 (44.3)
Housekeeper	55 (12)
Student	30 (6.5)
Others	171 (37.2)
**Education level**	
Illiterate	229 (49.8)
< Diploma	159 (34.5)
≥ Diploma	72 (15.7)
**Route of transportation**	
Ambulance	195 (42.4)
Private vehicle	265 (57.6)
**Intention**	
Intentional	13 (2.8)
Unintentional	447 (97.2)
**Location**	
Staircase	122 (27.3)
Scaffold	94 (21)
Roof top	37 (8.3)
Ladder	36 (8.1)
**Safety measures**	
Yes	422 (91.7)
No	38 (8.3)

**Table 2:** Clinical findings of studied patients when presenting to emergency department

**Variable**	**Number (%)**
**Vital Sign**	
Systolic blood pressure (mmHg)	111.4 ± 20.6
Respiratory rate (/minute)	17.2 ± 4.8
Pulse rate (/minute)	87.5 ± 15.3
O2 saturation (%)	95.4 ± 8.2
**Level of consciousness (GCS) **	
15	409 (88.8)
13-15	38 (8.3)
9-12	4 (0.9)
3-8	9 (2)
**Location of injury**	
Head and face	198 (43.1)
Spinal column	85 (18.5)
Upper extremity	85 (18.5)
Lower extremity	50 (10.8)
Thorax	24 (5.2)
Abdomen and pelvic	18 (3.9)
**Time of presentation**	
Morning (8 –14)	139 (30.2)
Evening (14 – 2)	295 (64)
Night (2 – 8)	27 (5.8)

GCS: Glasgow coma scale.

**Table 3:** Emergency department outcome of studied patients

**Variable**	**Number (%)**
**Discharge from ED**	293 (63.7)
**Admission to other wards**	153 (33.3)
**Transfer to other hospitals**	6 (1.3)
**Death**	8 (1.7)

ED: emergency department

Similar to the results of Zargar and Amani, most participants of this study had education level of less than high school diploma and were construction workers (4, 6). 

In the Amani et al. study, 29.3% of the patients were self-employed, 28.1% were unemployed , 17.3% were housekeepers, 10% had no job and 9.3 were construction workers (6). Considering that our study only evaluated a specific type of trauma, it was expected to mostly include construction workers and housekeepers. The route of transportation to hospital was private vehicle in most cases and was ambulance in only 42%. In the study in Isfahan, 49.6% of the patients were transported via ambulance, while in Ahwaz only 11.8% of the patients were transported via an ambulance (14, 15). Mean time of ambulance arriving and transportation to hospital were 16.9 and 27.11 minutes, respectively; which are similar to findings of Zamani et al. study (15). Head and face, spinal column and upper extremities were the most vulnerable body parts in this study, which is in line with the study by Hatamabadi et al. (16). However, in the Amani et al. study, head and face trauma was in second place after hands (6). In another study, head and face was in second place after extremities (15).

Outpatient treatment and discharge of trauma patients was reported to be more than 90% in Zargar et al. study (4). In victims of traffic accident in Abali road, outpatient treatment was more than 60%, hospitalization rate was 22.1%, rate of transfer to other hospitals was 1.5%, and discharge against medical advice was reported to be 16.1% (16). In another study, rate of discharge from ED was 49.3%, hospitalization in other wards was 43.2%, discharge against medical advice was 6.8% and death rate was reported as 0.7% (15).

 In total, fall from height is the second cause of trauma after traffic accidents in Iran, which confirms that these accidents should be considered and evaluated more and preventive measures such as using helmet, safety belt, and gloves should be mandatory for construction workers. Therefore, it is suggested to plan for further education and take more serious measures for these 2 types of trauma.

***Limitations and suggestions***

Since fall from height trauma patients were evaluated in 3 hospitals, namely Imam Hossein, Shohadaye Tajrish and Loghmane Hakim, it is possible that the participants do not fully represent the population of trauma patients in the society and it is suggested to carry out future studies in all hospitals around Tehran or even Iran. In addition, this study evaluates ED outcomes; therefore, the final outcome of the patients might be different than what has been reported. It is suggested to evaluate the outcome of the patients after discharge in a similar study. Considering the fact that many of the construction workers in this study had not used proper safety equipment for working in heights, it is suggested to plan for further education and supervision in this area.

## Conclusion:

Based on the findings, most fall from height victims in the present study were young men, single, construction workers, with less than high school diploma education level who were presented to ED following fall from a staircase or scaffold. Intentional fall and greater height of falling significantly correlated with mortality.

## Author contribution:

All authors passed four criteria for authorship contribution based on recommendations of the International Committee of Medical Journal Editors.

## References

[1] Jennett B (1996). Epidemiology of head injury. Journal of Neurology, Neurosurgery & Psychiatry.

[2] Ommaya AK, Thibault L, Bandak FA (1994). Mechanisms of impact head injury. International Journal of Impact Engineering.

[3] Watson WL, Ozanne-Smith J (2000). Injury surveillance in Victoria, Australia: developing comprehensive injury incidence estimates. Accident Analysis & Prevention.

[4] Zargar M, Zafarghandi M, Mdaghgh H, Abasi K, Rezai Shirazi H (1998). Significance of trauma mechanism and its effect on the outcome of trauma patients. Tehran University Medical Journal TUMS Publications.

[5] HATAMABADI H, SOORI H, VAFAEE R, HADADI M, AINY E, ASNAASHARI H (2012). Epidemiological pattern of road traffic injuries in Tehran-Abali Axis in 2008: A prospective study.

[6] Amani F, Habibzadeh S, Rostami K (2009). Specifications of traumatized patients referring to Fatemi hospital of Ardabil, 2007-8. Journal of Ardabil University of Medical Sciences.

[7] Zamani M, Esmailian M, Mirazimi MS, Ebrahimian M, Golshani K (2014). Cause and Final Outcome of Trauma in Patients Referred to the Emergency Department; a Cross Sectional Study. Iranian Journal of Emergency Medicine.

[8] Lehtola S, Koistinen P, Luukinen H (2006). Falls and injurious falls late in home-dwelling life. Archives of gerontology and geriatrics.

[9] Iida S, Hassfeld S, Reuther T, Schweigert H-G, Haag C, Klein J (2003). Maxillofacial fractures resulting from falls. Journal of Cranio-Maxillofacial Surgery.

[10] Tinetti ME, Speechley M, Ginter SF (1988). Risk factors for falls among elderly persons living in the community. New England journal of medicine.

[11] Scallan E, Staines A, Fitzpatrick P, Laffoy M, Kelly A (2001). Injury in Ireland.

[12] Tinetti ME (1994). Prevention of falls and fall injuries in elderly persons: a research agenda. Preventive medicine.

[13] Jeong BY (1998). Occupational deaths and injuries in the construction industry. Applied ergonomics.

[14] Salimi J ZM (2008). An epidemiologic study of trauma patients admitted to the Hospital Ahwaz Golestan. Payesh Quarterly.

[15] Zamani M, Esmailian M, Mirazimi MS, Ebrahimian M, Golshani K (2014). Cause and final outcome of trauma in patients referred to the emergency department: a cross sectional study. Iranian journal of emergency medicine.

[16] Hatamabadi H, Vafaee R, Haddadi M, Abdalvand A, Esnaashari H, Soori H (2012). Epidemiologic study of road traffic injuries by road user type characteristics and road environment in Irán: A community-based approach. Traf Inj Prev.

